# Frontiers in Mesoporous Nanomaterials

**DOI:** 10.3390/nano6010015

**Published:** 2016-01-11

**Authors:** Eva Pellicer, Jordi Sort

**Affiliations:** 1 Departament de Física, Universitat Autònoma de Barcelona, E-08193 Bellaterra, Spain; 2 Institució Catalana de Recerca i Estudis Avançats (ICREA) and Departament de Física, Universitat Autònoma de Barcelona, E-08193 Bellaterra, Spain

The Special Issue of *Nanomaterials* “Frontiers in Mesoporous Nanomaterials” gathers four reviews, one communication and eight regular papers. The prospective use of mesoporous silica in the biomedical field as drug delivery platform and antitumor agent is covered in the reviews by Wen and co-workers and Vallet-Regí and co-workers [1,2]. Likewise, application of mesoporous metal oxides in the energy field and, in particular, in supercapacitors, is also reviewed by Yang and co-workers [3]. Finally, the review by Choi and colleagues takes a more fundamental-oriented look into various MCM-41 silica-based materials and their conducting nanocomposites employed in electro-responsive electrorheological fluids [4]. The interest that mesoporous materials are arising in many other fields like electrocatalysis, gas-sensing or tissue regeneration is also represented in this Special Issue. Given the widespread range of applications, researchers have continued dedicating efforts in searching new, facile, low-cost synthetic routes toward novel mesoporous siliceous and non-siliceous materials and exploring the effect of synthetic parameters over their morphological and microstructural features, long-range order, *etc*. An overview of various approaches towards the synthesis of these innovative materials can be found in this Special Issue.

Since their discovery in the 90’s, interest in mesoporous silica materials continues nowadays intact [5]. For example, Pluronic F127 surfactant was utilized by Li *et al.* [6] to prepare a series of mesoporous silicas and mesoporous organosilicates with hierarchical porosity through evaporation-induced self-assembly (EISA). Two silica sources, tetraethyl orthosilicate (TEOS) and triethoxysilane hydrosilylated octavinyl polyhedral oligomeric silsesquioxane (OV-POSS-SILY), were employed at variable weight ratios in order to tune the mesophase of the resulting mesoporous silica. Ordered body-centered cubic (bcc) structure (for TEOS alone), ordered face-centered cubic (fcc) structure (for 10 and 20 wt % OV-POSS-SILY), and disordered spherical pores (for ≥30 wt % OV-POSS-SILY) were obtained. According to the authors, the porosity enhancement brought by the cage-like POSS within the silica walls together with the remaining vinyl groups from the OV-POSS-SILY units, make these mesoporous organosilicas appealing as low-k interlayer dielectric materials and as catalysts.

In the review by Choi and co-workers, the synthesis and electro-responsive properties of smart fluids containing mesoporous particles, with special emphasis on MCM-41 silica and conducting polymer-modified MCM-41 composite suspensions, is reported [4]. The mesoporous particles show electrically-tunable viscoelasticity when dispersed in an insulating liquid. Polypyrrole (PPy) and copolyaniline (COPANI) are given as examples of polymers in combination with the mesoporous silica particles. The main feature of these smart fluids relies on the reversible and tunable phase transition from liquid-like to solid-like state upon application of an external electric field (denoted as electrorheological phenomenon). For this reason, they have potential applications in a variety of active control systems (dampers, clutches, shock absorber systems, microfluidics, ER polishing, ER haptic device, and tactile displays).

Current mechanisms of mesoporous silica nanoparticle (MSN)-based responsive controlled drug release systems are reviewed by Wen and co-workers [1]. The different stimuli: redox reaction, pH, light, temperature, magnetic field, biomolecule-assisted (and combinations thereof), and free-blockage are analysed by pointing its main landmarks. The rapid evolution of smart nano-switches has prompted the upgrading of controlled release systems from single input switching to reversible, multifunctional, complicated logical switches and selective switches. In particular, the free-blockage switches, which are based on hydrophobic/hydrophilic conversion, have been proposed and designed in the last two years. The various strategies aimed at providing MSNs with active targeting are extensively reviewed by Vallet-Regí’s group [2]. In addition, the different means by which pore opening in MSNs is triggered and the entrapped cargo is released once the MSNs are located at the target tissue are also re-examined. Taking into account that MSNs are proposed as substitutes to conventional chemotherapy, key issues like safety, tissue accumulation, and elimination of MSNs are outlined as well in this insightful review.

The prospective use of IBN-4 mesoporous silica nanomaterials as enzyme carriers for prodrug therapy is investigated by Hung *et al.* [7]. Horseradish peroxidase (HRP) was immobilized onto the mesoporous silica nanoparticles through covalent bonds. The ability of the HRP-encapsulated IBN-4 to convert a prodrug (indole-3- acetic acid (IAA)) into cytotoxic radicals is tested with good results. These radicals are shown to be able to trigger tumor cell apoptosis in human colon carcinoma (HT-29 cell line) cells without damaging their membranes.

Besides its prominent use as drug delivery platforms, mesoporous materials also hold promise in tissue engineering. For example, hierarchically porous Poly-l-lactic acid (PLLA) scaffold can be fabricated via two-step thermally induced phase separation (TIPS) process and gelatin fibers can then be introduced into the pores of PLLA [8]. The grid-like distribution of gelatin on the PLLA pore walls increases the specific surface area, hydrophilicity, mechanical integrity and protein adsorption capacity of the material. Human adipose tissue-derived stem cells (ADSCs) are seeded into the resulting 3D PLLA/gel network structure in order to assess cell morphology, viability and osteogenic differentiation. The results show that the ADSCs are able to largely proliferate, which indicates that the *in vitro* constructed biomimetic scaffolds show good cell compatibility, and could be further transplanted to the body to repair bone damaged tissue.

Mesoporous alumina membranes are widely utilized as templates for the growth of zero-dimensional (nanoparticles) and one-dimensional (nanowires and nanorods) materials, as well as in nanofluidics, sensors, and drug delivery applications. Understanding the diffusive transport of molecules or ions through a nanochannel or nanopore associated to a concentration difference of the solutions at both channel ends is essential for the aforementioned uses. Having this in mind, the diffusive transport of Na^+^ and Cl^−^ ions within the channels of alumina membrane synthesized by two-step anodization is reported by Benavente’s group [9]. Salt diffusion measurements performed within a wide range of NaCl concentrations indicate a reduction of ~70% in the value of the NaCl diffusion coefficient through the membrane pores with respect to the bulk of the solution. Accordingly, the authors conclude that the minimum NaCl concentration as the feed solution necessary to neglect the contribution of electrical interactions in the diffusive transport is 0.05 M.

Regarding non-siliceous mesoporous materials, transition metal oxides cope much of the interest within the last twenty years [10]. In [11], Tiemann’s group conduct a thermogravimetric analysis of the thermally induced processes during the nanocasting synthesis of various metal oxides by using mesoporous CMK-3 carbon as the template. The authors find that the metal nitrates dispersed in the pores of CMK-3 carbon tend to decompose at lower temperature than in a non-confined scenario. Moreover, the temperature interval for the thermal decomposition of the CMK-3 carbon matrix also varies significantly, depending on which metal nitrate is used. In any case, the onset temperature is always lower than for the bare metal nitrate-free CMK-3 carbon. It is believed that the as-formed finely dispersed metal oxides catalyze the combustion, which results in lower combustion temperature. A strong catalytic effect is observed for the oxides of transition group VII and VIII metals (Mn, Fe, Co, Ni), as well as of Cu and Ce.

Recent progress in the synthesis and electrochemical performance of mesoporous transition metal oxides typically used as electrodes for electrochemical capacitors (*i.e.*, supercapacitors), RuO_2_, MnO_2_, NiO, Co_3_O_4_ and NiCo_2_O_4_, is summarized by Yang and co-workers [3]. The highest specific capacitances reached so far, either alone or in the form of composites, are indicated. These large values arise from the effective contacts between electrode materials and electrolytes and the fast transportation of ions and electrons within the bulk of the electrode and at the electrode/electrolyte interface.

Mesoporous MnO_2_ is perhaps one the transition metal oxides currently gathering more attention for its use as electrode material in supercapacitors. Compared to ruthenium oxide and cobalt hydroxide, MnO_2_ shows lower electrical and ionic conductivity, which has hampered its massive implantation in energy storage devices. However, providing mesoporosity to the material is proven to enhance these features since it brings an increase in the specific surface area. In the work by Jiang *et al.* [12], mesoporous MnO_2_ is synthesized by reacting potassium permanganate with ethylene glycol and further mixing the resulting material with Ag nanowires. At the same time, conventional Na_2_SO_4_ electrolyte is replaced by KI as it has been suggested that the total capacitance and energy density of supercapacitors can be greatly improved if an electrochemically-active electrolyte is employed. Compared to mesoporous MnO_2_, mesoporous MnO_2_/Ag nanowires composite shows five times larger current (0.5 A g^−1^ over 0.1 A g^−1^) as well as higher specific capacitance in the KI electrolyte. Results are also much better than in Na_2_SO_4_ electrolyte.

The synthesis of mesoporous CuO/CeO_2_ composite materials by nanocasting from SBA-15 silica and metal nitrates as precursors is reported in the work by Markoulaki *et al.* [13]*.* The authors investigate the morphological, structural and textural characteristics of a series of mesoporous CuO_(x)_/CeO_2_ materials prepared with variable loading amounts of Cu(NO_3_)_2_·5H_2_O. Hexagonally-packed nanorods with BET surface areas in the range from 83 to 142 m^2^ g^−1^ are reported. The obtained materials are tested as electrolyzers for the UV-Vis light-driven oxygen evolution reaction (OER) with good results. The best performing sample toward OER contains ~38 wt % CuO, for which the O_2_ evolution rate is ~392 μmol h^−1^ g^−1^ with a QE of 17.6% at λ = 365 ± 10 nm and incident photon conversion efficiency of 1.3% in the 360–780 nm range.

Novel rose-like WO_3_ hierarchical architectures are shown to be successfully synthesized through a hydrothermal method using oxalic acid. According to the author [14], the resulting 3D structures are constructed by numerous nanosheets that assemble together leaving numerous mesopores beneath. As a result, the material shows a moderate specific surface area (78 m^2^ g^−1^). Gas-sensing measurements reveal that the rose-like WO_3_ material can detect CO gas, outperforming conventional WO_3_ powders and films.

Compared to metal oxides, the synthetic methodologies toward mesoporous metals and alloys have been much less explored. Current trend indicates that this is indeed an emerging topic [15]. Lyotropic liquid crystals (LLC) self-assembled metal nanoparticle (NP) frameworks is an innovative synthetic approach to stabilize mesoporous metal frameworks. In [16], the block co-polymer Pluronic F127 was used as sacrificial template to form mesoporous metal structures by incorporating Ag, Ni and Cu metal NPs into the hydrophilic domain of the ordered LLC matrix. The key feature of this approach relies on the fact that Pluronic F127 can arrange into various ordered or semi-ordered long-range order structures, including lamellar, cubic, or hexagonal structures when dissolved in water at appropriate concentrations. The synthetic pathway consists in the following steps: (1) the dispersion of the LLC with the NPs in water; (2) the casting of the resulting solution or gel onto a silicon wafer by dip and spin coating; (3) the thermal annealing of the thin film in air to remove the LLC. The impact of the annealing temperature on the stability of the mesostructure upon LLC removal is investigated by the authors in an attempt to correlate porosity formation with the mechanism of coalescence. Clustering of the NPs via thermal sintering when carbonizing the samples, and collapse of the initial particle distribution, unavoidably occurs.

In summary, this Special Issue brings a compilation of articles that brilliantly illustrates, on the one hand, the sustained efforts that researchers devote on devising novel, optimized synthetic routes and, on the other hand, the enormous potential that mesoporous materials hold in diverse technological fields.
